# Seafood Allergy in Asia: Geographical Specificity and Beyond

**DOI:** 10.3389/falgy.2021.676903

**Published:** 2021-07-08

**Authors:** Christine Y. Y. Wai, Nicki Y. H. Leung, Agnes S. Y. Leung, Gary W. K. Wong, Ting F. Leung

**Affiliations:** ^1^Department of Paediatrics, Faculty of Medicine, The Chinese University of Hong Kong, Hong Kong, China; ^2^Hong Kong Hub of Paediatric Excellence, The Chinese University of Hong Kong, Hong Kong, China

**Keywords:** prevalence, tropomyosin, parvalbumin, Crustacea, mollusk, fish

## Abstract

Asian countries have unique ways of food processing and dietary habits that may explain the observed differences in the prevalence, natural history, epidemiology and sensitization pattern of food allergic diseases when compared to western countries. Per capita consumption of seafood, including fish and shellfish, is well above the global average for many Asian countries because of their coastal geographical location and rich seafood supply. The wide availability and high abundance of seafood in Asian countries have shaped a diverse way of processing and eating this major food group. Such unique features have significant impact on the sensitization profile and allergenicity of Asians to fish and shellfish. For example, fish and shellfish are eaten raw in some countries that may promote sensitization to heat-labile allergens not otherwise seen in other regions. Fermented fish sauce is commonly used as a condiment in some countries which may promote fish sensitization. Shrimp head and shrimp roe are regarded as delicacies in some countries, but their allergen profiles are yet to be characterized. Freshwater fish and shellfish are a common food source in many Asian countries but the allergenicity of many such species remains unknown. In this review, we discuss factors that may contribute to differences in molecular profile and sensitization pattern for fish and shellfish that are observed in Asian populations and revisit the current status of seafood allergy in this part of the world. Acknowledging the similarities and differences of seafood allergy patterns between Asian and western populations can help us refine a better strategy for diagnosing and managing seafood allergy.

## Introduction

Food allergy is a common allergic disease that affects up to 10% of the population, and its prevalence has been increasing over the past decades (1, 2). While the “big eight” food groups account for 90% of reported allergic reactions (3), the most common triggers of food allergic reactions in a particular geographical location are largely influenced by the feeding pattern, dietary habit and food availability (4). Per capita consumption of seafood, including fish and shellfish, is much higher in Asia when compared to the global average. Asian countries consumed two-thirds of global seafood production (5). In light of this, it is not surprising that seafood allergy is of high importance in Asia where its prevalence was up to 7.7% in some countries (6). Furthermore, Asian countries have distinct ways of seafood processing and dietary habits, which may account for substantial differences observed in the natural history and sensitization profile of seafood allergic subjects when compared to western countries. For example, fish is introduced early into the infants' diet that parallels the younger age at onset of fish allergy in Asian children (7). Seafood is often consumed raw in Asian cuisines, and this eating practice can promote sensitization to heat-sensitive seafood allergens which is not reported in western countries. Besides, Asians consume some seafood species that are not being consumed in other parts of the world. With the advancement of molecular allergology, we are now able to examine allergen components in depth. Emerging studies have reported the occurrence of monosensitization to particular seafood species in this region. In this review, we discuss seafood allergy from the Asian perspective. Acknowledging important differences between East and West and unique features of seafood allergy in Asia would provide important insight on the clinical management of seafood allergy.

## Prevalence and Natural History of Seafood Allergy in Asia

### Prevalence of Shellfish Allergy in Asian Countries

Shellfish is one of the most common food allergen sources in Asia (Table 1), and the leading cause of food allergy in Taiwan (18, 19), Thailand (20), Singapore (21), Vietnam (9, 12) and Hong Kong (22). In a survey in which IgE sensitization and skin prick tests were used to determine food allergy prevalence, shrimp was found to be the most commonly sensitized food allergen source in Hong Kong, China and India (8). Up to 13.1 and 10.3% of the Chinese and Indian populations had shrimp-specific IgE (sIgE) that exceeded 0.70 kUA/L, while over 2% of the Hong Kong population was positive to shrimp by skin prick test. On the other hand, shellfish allergy is less common in East Asia where shellfish only accounted for 3.4% of all reported food allergy cases in Japan (23) and 0.87% of parent-reported shellfish allergy in South Korea (13). The prevalence of shellfish allergy was affected by environmental exposures, dietary habits and cross-sensitization with other arthropods such as dust mite or cockroaches (24, 25).

**Table 1:** Population-based survey on the prevalence of seafood allergy in Asian countries over the past 10 years.

**References**	**Location**	**Sample size**	**Age range (Year)**	**Methodology**	**Shellfish (rank)**	**Fish (rank)**
Li et al. (8)	Hong Kong	6,194	6–11	sIgE > 0.7 kUA/L	4.7% (3rd)	1.2% (21st)
				SPT	2.43% (1st)	0.22% (4th)
				sIgE + symptoms	1.05% (1st)	0.2% (2nd)
	Guangzhou	5,542	6–11	sIgE > 0.7 kUA/L	2% (2nd)	0% (/)
				SPT	1.14% (1st)	0% (/)
				sIgE + symptoms	0.18% (1st)	0% (/)
	Shaoguan	5,139	6–11	sIgE > 0.7 kUA/L	13.1% (1st)	0% (/)
				SPT	4.53% (1st)	0.04% (10th)
				sIgE + symptoms	0.65% (1st)	0% (/)
	Bengaluru/Mysore	5,677	6–11	sIgE > 0.7 kUA/L	10.3% (1st)	0.4% (26th)
				sIgE + symptoms	0% (/)	0% (/)
Le et al. (9)	Vietnam	9,039	16–50	Self-reported	6.88% (1st)	3.71% (2nd)
				Doctor-diagnosed	2.95% (1st)	1.58% (2nd)
Dai et al. (10)	Wenzhou	4,151	3–6	Self-reported	2.46% (2nd)	1.35% (6th)
Ziyab et al. (11)	Kuwait	3,864	11–14	Questionnaire + Clinical history	1.3% (5th)	1.6% (3rd)
Le et al. (12)	Hue	4,443	2–6	Self-reported	5.22% (1st)	1.55% (2nd)
				Doctor-diagnosed	4.79% (1st)	1.37% (2nd)
	Tien Giang	4,177	2–6	Self-reported	4.29% (1st)	1.7% (4th)
				Doctor-diagnosed	2.8% (1st)	1.1% (4th)
Kim et al. (13)	Korea	29,842	6–16	Self-reported	0.84% (2nd)	0.32% (3rd)
Park et al. (14)	Seoul	16,749	0–6	Self-reported	0.5% (5th)	0.4% (6th)
Dey et al. (15)	Kolkata	5,161	All	Self-reported	3.43% (/)	4.59% (/)
Lao-araya et al. (16)	Chiang Mai	452	3–7	Self-reported	3.32% (2nd)	1.1% (4th)
Ho et al. (17)	Hong Kong	7393	0–14	Self-reported	1.79% (1st)	0.19% (8th)
Wu et al. (18)	Taiwan	30,018	All	Self-reported	7.23% (1st)	1.32% (2nd)

### Prevalence of Fish Allergy in Asian Countries

Fish allergy is less common in Asia than shellfish allergy, but its prevalence is still considerably high (Table 1). Fish allergy affected 1.55–1.60% of Vietnamese, which was second to shellfish as the most common type of food allergen source (9, 12). The prevalence of self-reported fish allergy was 2.29% in Philippines where fish is part of the weaning diet and fish sauce widely used as a condiment (26). In contrary, parent-reported fish allergy affected <1.5% of other Asian populations (11, 13, 22, 26). The rates of IgE sensitization to fish were even lower, when none of the 10,681 subjects in China was positive for fish-specific IgE and only 1.2% of subjects in Hong Kong were sensitized to fish (27). Nevertheless, this study only selected cod as the benchmark for sIgE, when this fish species is not popular in many Asia populations. Thus, results from this study probably underestimated the prevalence of fish allergy in Asia.

### Natural History of Shellfish Allergy in Asia

Seafood allergy is generally believed to be persistent throughout life despite limited published evidence on its natural history (28). Shellfish allergy had late onset at a median age of 25 years old in Canadians (29), whereas shellfish allergy started much earlier in the Asian population. While shellfish allergy affected 0.1% of children aged 0–5 years in the United states (30), IgE sensitization to shellfish was as high as 10.6% among Singaporean children younger than 3 years of age (31). It was noteworthy that shellfish was the most frequent cause of food allergy in several studies that involved school-age or pre-school Asian children (9, 12, 18–22), which was probably attributed to the early introduction of shellfish in the Asian diet. Another possible reason is that exposure to other arthropods like dust mite or cockroach in tropical or subtropical areas of Asia may increase the shellfish allergy through cross-reactive allergens. Shellfish allergy also tends to persist longer as shellfish was reported to be the leading cause of food-induced anaphylaxis in older children and adults in Singapore (32), Hong Kong (33) and Thailand (34). Nevertheless, a Thai study found that 46% of shrimp-allergic patients were able to tolerate shrimp 10 years later, suggesting that some individuals could outgrow shrimp allergy (35). In addition, Ayuso et al. reported greater epitope recognition for shrimp in children than adults, supporting that shrimp sensitization could decrease with age (36). It remains unclear at this stage whether this is a general observation or is limited to some populations.

### Natural History of Fish Allergy in Asia

Similar to shellfish allergy, it is generally believed that fish allergy persists throughout life although the relevant evidence is limited. One study suggested that 65.5% of fish-sensitized infants maintained their sensitization at school age (37). Another study reported that the prevalence of fish allergy was slightly higher in American adults (0.9%) than children (0.6%) (38). The same study also found that nearly 40% of adults having fish allergy started to suffer from this allergy in adulthood. Similar findings were reported in Philippines (26). Alternatively, a small study reported that sIgE levels and skin prick test positivity of fish-allergic patients diminished over a 9-year period, and some subjects were able to tolerate fish with lower allergenicity such as tuna and swordfish (39). Longitudinal studies are required to elucidate the natural history of fish allergy. It is also noteworthy that allergenicity of different fish species could vary significantly, and it is important to distinguish between fish-allergic patients with partial tolerance and true resolution of their fish allergy.

### Shellfish and Fish Allergen Sources and Allergens

#### Molecular Characteristics of Shellfish Allergens

The filamentous muscle protein tropomyosin was first identified as a shrimp allergen in 1993 (40), which was subsequently reported to be a pan-allergen among invertebrates including crustaceans, arachnids, insects and mollusks (41). Tropomyosin is a heat-stable allergen that can withstand high temperature, food processing methods including ultrasound and gamma irradiation, and gastric digestion (42). Arginine kinase was identified as another pan-allergen but, in contrast to tropomyosin, this heat-labile protein exhibited reduced IgE reactivity upon thermal and pH treatment (43). Myosin light chain, sarcoplasmic calcium-binding protein, troponin C, triosephosphate isomerase, and fatty acid-binding protein are other shellfish allergens being registered in the WHO/IUIS allergen database, although their physiochemical properties remain largely unknown (44, 45). Paramyosin was identified as an allergen in mollusk that have limited cross-reactivity with allergens in crustaceans (46).

#### Molecular Characteristics of Fish Allergens

The calcium-binding muscle protein parvalbumin was first identified as the major fish allergen more than 50 years ago (47). It is highly conserved across all bony fishes (48). Similar to tropomyosin, parvalbumin was resistant to thermal treatment and enzymic hydrolysis (47). Despite high homology across fish species, parvalbumin had different allergenicity in different fish species (48), and the allergenicity of individual fish species was also dependent on the quantity of parvalbumin present in the muscle (49). Collagen was identified as the second heat-stable fish allergen from muscle and skin of fishes (50, 51), but its allergenicity and characteristics were only studied in a small number of fish species. Two heat-labile muscle enzymes of fish, aldolase and enolase, were recently identified as allergens but their clinical relevance remained unclear (52). Tropomyosin was also registered as a fish allergen in the WHO/IUIS database, although its allergenicity has only been demonstrated in one fish species, the Mozambique tilapia. Apart from muscle proteins, fish roe was also reported to cause allergic reactions due to the presence of yolk protein vitellogenin (53).

#### Major Shrimp Allergens in the Asian Population

Seawater shrimps such as *Penaeus monodon* and *Litopenaeus vannamei* are the most widely distributed and marketed shrimps in the world, and their respective allergen profiles have been well-characterized (Table 2). Freshwater shrimps are also popularly consumed in Asia, including the giant freshwater prawn *Macrobrachium rosenbergii* in Thailand and Taiwan and the freshwater Siberian prawn *Exopalaemon modestus* in China, Korea and Taiwan. Tropomyosin was identified as the major shrimp allergen, and tropomyosins of freshwater and seawater shrimps shared over 95% sequence homology (54, 55). Arginine kinase and glyceraldehyde 3-phosphate dehydrogenase (GAPDH), on the other hand, were identified as major shrimp allergens in *M. rosenbergii*, with arginine kinase being cross-reactive with *Gryllus bimaculatus* (field cricket) (56, 57). Interestingly, the oxygen-transport protein hemocyanin was found to be a species-specific allergen in *M. rosenbergii*. Hemocyanins of tiger shrimp and mud crab was unable to inhibit IgE binding to *M. rosenbergii* hemocyanin (58), while a Thai study reported isolated sensitivity to *M. rosenbergii* based on results of oral food challenges (59).

**Table 2:** Summary of shellfish and fish allergens.

**Allergen source**	**Allergen**	**Molecular weight (kDa)**	**Body part**
Shellfish	Tropomyosin	34–38	Muscle
	Arginine kinase	40–45	
	Sarcoplasmic calcium-binding protein	20–25	
	Myosin light chain	17–20	
	Troponin C	21–21	
	Triosephosphate isomerase	28	
	Fatty acid-binding protein	15–20	
	Paramyosins	100	
	Myosin heavy chain	225	
	Pyruvate kinase 2	58	
	Filamin C	90	
	Hemocyanin	60–80	Cephalothorax
	Glyceraldehyde 3-phosphate dehydrogenase (GDPH)	37	Shell
	Vitellogenin	282	Ovary
	Ovarian peritrophin 1 precursor	30	
	β-actin	41–46	
	14-3-3 protein	27	
Fish	Parvalbumin	10–12	Muscle
	Enoloase	50	
	Aldolase	40	
	Collagen	130–140	
	Tropomyosin	32	
	Lipovitellin	150–160	Roe
	β'-component	16–35	

#### Major Crab Allergens in the Asian Population

Crab is another popular crustacean food item worldwide. In China, crab was the most common culprit for severe adverse food reactions caused by shellfish (8). Crab was also the most common food allergen source among Taiwanese schoolchildren while 16.2% of Singaporean adults had crab allergy (60). The more commonly consumed crabs in western countries include blue crab (*Callinectes sapidus*), snow crab (*Chionoecetes opilio*), Dungeness crab (*Cancer magister*), edible crab (*Cancer pagurus*) and king crab (*Paralithodes camtschaticus*) (21, 61), while Chinese mitten crab (*Eriocheir sinensis*), mud crabs (*Scylla paramamosain, Scylla serreta*, and *Scylla tranquebarica*), red crab (*Charybdis feriatus*) and blue crab (*Portunus pelagicus*) were more popularly consumed in Asia. The IgE-binding proteins of these latter crab species have been extensively studied (62–66). Unlike shrimps, both tropomyosin and arginine kinase are major allergens in crab (Table 2). Arginine kinase is a 41 kDa phosphagen kinase that plays an important role in cellular energy metabolism in invertebrates. Apart from these two major allergens, sarcoplasmic calcium-binding protein, triosephosphate isomerase, pyruvate kinase 2, myosin light chain I and filamin C were minor crab allergens (56, 67, 68). Jasim et al. (69) identified three common crab allergens including tropomyosin, arginine kinase and actin as well as a novel allergen hemocyanin from *S. transquebarica* that is commonly consumed in Malaysia.

#### Major Mollusk Allergens in the Asian Population

Besides crustaceans such as shrimp and crab, mollusks are popular food item in Asian countries. There is a common belief that mollusks offer health-promoting effects because of their rich vital nutrients and active secondary metabolites (70). Cross-reactivity between crustaceans and mollusks were well-recognized (42). Despite this, Suzuki et al. (46) identified a 100 kDa myofibrillar protein paramyosin in the disc abalone *Haliotis discus* as a novel allergen. This protein reacted with 16 of the 18 Japanese sera from allergic subjects being studied. They also reported IgE reactivity against paramyosins from turban shell, mussel, scallop, squid and octopus, which was subsequently found in the Asian rapa whelk *Rapana venosa* (71). Interestingly, paramyosin is a heat-labile protein so the clinical relevance of paramyosin would be limited to some Asian populations such as Japanese and Koreans where mollusks and abalone are served as sashimi.

#### Major Echinoderm Allergens in Asian Population

Although phylogenetically distinct from crustaceans, mollusks and fin fish, echinoderms including starfish, sea cucumbers and sea urchins are also popular seafood delicacies in China, Japan and Korea. For instance, Japanese consume eggs of starfish and sea urchin roe called “*uni*.” Worldwide consumption of sea urchin has been increasing, and several case reports of anaphylaxis to sea urchin roe or upon stinging by starfish were published (72, 73). A 118-kDa protein in sea urchin roe was presented as a potential allergen by immunoblotting, which was identified as major yolk protein by MALDI-TOF mass spectrometry (74).

### Insect Consumption and Cross-Reactivity With Shrimp

Insects have become a unique diet in China under the cultural influence of traditional Chinese food and medicine. The consumption of oil-fried, water-boiled or ground silkworm pupa is common in China whereas silkworms have been used as therapeutics in traditional medicine and also commonly consumed after boiling with soybean sauce in Korea. Although insects are not widely consumed in western countries at present, edible insects have gained attention as a novel and sustainable food for protein sources worldwide due to the worry about food scarcity.

However, there is increasing recognition about allergy to insects. From 1980 to 2007, 61 (17%) of 358 reported cases of anaphylaxis were triggered by insects including silkworm pupas, cicada pupas, grasshoppers, locusts and hawkmoth (75). Over 1,000 patients experienced anaphylactic reactions following consumption of silkworm pupa annually in China, and foreign tourists also experienced anaphylactic shock after eating silkworm (76). Considering tropomyosin as a pan-allergen in invertebrates including crustaceans, mollusks, dust mites and cockroaches, shrimp-allergic patients are at risk of insect allergy due to cross-reactivity between allergens. As early as 1996, Leung et al. reported co-sensitization among insects, cockroach, fruit fly and shrimp (77). More recently, Jeong et al. (78) reported among 15 patients diagnosed with silkworm pupa allergy that 11 (73.3%) exhibited sIgE reactivity to shrimp and crab by ImmunoCAP (≥0.35 kUA/L) and 53.3% of patients' sera reacted to silkworm pupa tropomyosin. Kamemura et al. (79) also demonstrated strong positive correlation between shrimp- and cricket-specific IgE responses by the IgE crosslinking-induced luciferase expression (EXiLE) assay. Similarly, Broekman et al. (80) showed that all 15 shrimp-allergic patients were also sensitized to mealworm. There was similar basophil reactivity of these shrimp-allergic patients to all tested insect species including mealworm, cricket, grasshopper and moth. By means of immunoblot and nano LC-MS/MS analyses, insect extracts were found to contain a considerable number of arthropod allergens including tropomyosin, arginine kinase, myosin light chain and triosephosphate isomerase. These allergens probably accounted for cross-reactivity between shrimp and insects. Therefore, some shrimp-allergic subjects may also be allergic to insects.

#### Major Fish Allergens in Asian Population

Unlike shrimp and crab where regional availability largely governs the species consumed, many fish species such as cod, salmon and tuna were distributed and consumed worldwide. In addition to these imported species, many inland Asian countries rely on freshwater aquaculture. Freshwater fishes such as carp, tilapia and catfish are stable food sources in many Asian populations. Freshwater fishes usually have a muddy taste and contain bacteria and parasites that impose food safety concern when they are eaten raw. Heat-labile fish allergens such as enolase and aldolase may thus be less relevant in allergy to freshwater fishes (Table 2). Our team has recently identified parvalbumin as the major allergen *Cten i* 1 in grass carp (*Ctenopharyngodon idella*) using sera from 69 subjects with IgE-mediated fish allergy (81). *Ctenopharyngodon idella* is the most commonly consumed freshwater fish species in Hong Kong, which is phylogenetically close to the common carp (*Cyprinus carpio*). By inhibition ELISA, we neatly illustrated stronger IgE inhibitory effect against *Cten i* 1 by *Cyp c* 1 (parvalbumin from common carp, ~80% inhibition) when compared to *Gad m* 1 (parvalbumin of codfish) and *Sal s* 1 (parvalbumin of salmon). These findings could be explained by stronger allergenicity for parvalbumin of freshwater fishes than seawater fishes and/or stronger avidity of IgE to freshwater fish parvalbumin. On the other hand, Reuthers et al. (82) analyzed the IgE reactivity of 16 freshwater fishes available in Vietnam with sera from 18 Australian and three European patients with history of fish allergy. Concordantly, parvalbumin was found to be the major allergen in all freshwater fishes. Several isoforms of parvalbumin were detected in most fish species.

Apart from parvalbumin, Liu et al. (83) detected IgE binding in 10 proteins sized from 17 to 114 kDa from tilapia (*Oreochromis mossambicus*). The sensitization rate of subjects to these proteins ranged from 20 to 80%. All patients reacted a 32 kDa-protein which was identified as tropomyosin (*Ore m* 4) by LC-ES-MS/MS. *Ore m* 4 shared 58.8% sequence similarity as tropomyosin from northern shrimp, but 87.7% homology with human tropomyosin isoform 5. It should be noted that any protein resembling human proteins appear to have limited allergenic potential. In this patient group, 6 of 10 subjects suffered from inflammatory bowel disease (IBD). It is possible that anti-tropomyosin IgE antibody cross-reacted with gut proteins to result in IBD. It also raised a query if tropomyosin is a clinically relevant allergen in fish allergy.

## Effect of Unique Dietary Habits in Asian Population to Allergen Sensitization Profile

### Raw Fish Consumption and Collagen Sensitization

Collagen was first identified as a fish allergen in a Japanese study (50), and subsequently regarded as a pan-allergen in this population. Half of 36 fish-allergic Japanese patients reacted to collagen extracted from Pacific mackerel, in comparison to only 16 (44%) patients who were positive to parvalbumin (84). In addition, 13 (36%) subjects were mono-sensitized to collagen. Collagen from Pacific mackerel also significantly inhibited IgE binding to heated crude extract of 22 fish species by 87–93%. In contrast, the sensitization rate to collagen was low in European and Australian populations (52, 85). This much higher rate of collagen sensitization together with strong IgE inhibition in Japanese might be explained by their traditional dietary practice of eating raw fish sashimi or sushi. The risk of collagen sensitization was low in cooked fish, while the insoluble intact collagen found in raw fish might be a potent sensitizing allergen due to its resistance to gastric acid digestion (84).

### Fish Roe Consumption and Vitellogenin Sensitization

Fish roes are nutritious food commonly consumed in Japan. Salmon roe, also known as salmon caviar, red caviar, rainbow trout roe and ikra, is a common Japanese cuisine. Fish roe is the fifth most common food allergen source in Japan, and it is mandatory in the Japanese food sanitation law to label fish roe as a possible food allergen. In a retrospective study, half of 68 Japanese subjects reacted to salmon roe on oral food challenge (86). Analysis of sera collected from 20 patients with salmon roe allergy found that β'-component (β'-c) was the major allergen (53). Nine (45%) of 21 sera reacted to lipovitellin. Both lipovitellin and β'-c are degradation fragments of vitellogenin which issynthesized in fish liver and carried to oocytes through bloodstream. β'-c was also characterized as a major fish roe allergen in teleostean such as large yellow croaker (*Pseudosciaena crocea*) that is widely consumed in China (87). In the contrary, fish roe allergy is rare in western countries. When there is popular consumption of fish roes from sturgeon, paddlefish, cod, lumpfish, capelin, herring and salmon worldwide, there were increasing number of cases with fish roe allergy in USA, Portugal, Spain and Finland (88–91).

### Crustacean Body Parts Consumption Leading to Diversified Allergen Sensitization

Over the past decades, we found more reports on the profiling and characterization of allergens from shrimp as a prototype for shellfish. Tropomyosin has long been regarded as a major crustacean allergen. Over 80% of European shellfish-allergic subjects were sensitized to tropomyosin (92, 93). However, our recent study suggested that only slightly over half of subjects with challenge-proven shrimp allergy were sensitized to tropomyosin (94). Such low sensitization rate to tropomyosin was also reported in Thai (34.2%) and Japanese (37%) (95, 96), supporting that tropomyosin might not be the major allergen in Asians. We further demonstrated that Chinese patients were sensitized to multiple shrimp allergens, with troponin C and fatty acid-binding protein being the major allergens (97, 98).

One possible reason for such diversified sensitization profiles in different Asian populations might be due to the consumption practice for non-muscle body parts of shrimp and crab. While western populations mainly consume muscle of shelled shrimps, shrimp is always served head-on and shell-on in East and Southeast Asia when the shell, cephalothorax (containing brain, heart, stomach and bladder), ovaries and hepatopancreas are consumed together with shrimp muscle. The best example is hemocyanin that is found mainly in shrimp cephalothorax but not muscle. In addition, Khanaruksombat et al. (99) reported novel allergens from muscle, shell, hepatopancreas and ovaries of banana shrimp, *Fenneropenaeus merguiensis*, that is commonly consumed in Thailand. They detected four immunoreactive bands from shell extract and six immunoreactive bands from ovarian extracts at different vitellogenic stages. On top of arginine kinase and sarcoplasmic calcium-binding protein, these investigators identified a novel allergen called glyceraldehyde-3-phosphate dehydrogenase from the shell. Interestingly, different allergens including vitellogenin, ovarian peritrophin 1 precursor, β-actin and 14-3-3 protein were detected at different stages of ovarian development. It is noteworthy that vitellogenin is a female specific protein also found in the hemolymph of most crustacean species, and its degradation fragments lipovitellin and β'-c were defined as major fish roe allergens. This study highlighted the presence of IgE-binding proteins in both shrimp muscle and organs.

### Fermented Shrimp Processing and Its Effect on Shrimp Allergenicity

Fermented foods such as kimchi and *saeujeot*, a salted and fermented shrimp product, are popular food items in Korea and some other Asian countries. *Saeujeot* is ripened primarily by proteolytic enzymes present in shrimp, and this process confers characteristic flavor and aroma in this food item. Although being heat stable, the allergenicity of tropomyosin decreased during fermentation of shrimps. Park et al. (100) demonstrated that tropomyosin could not be detected after 6 days of fermentation at 25°C, 10 days at 15°C and 30 days at 5°C. More importantly, the binding ability of tropomyosin in *saeujeot* to patient sera decreased gradually during fermentation process. This binding ability decreased further and faster at higher fermentation temperature, which might be caused by the activity of a trypsin-like enzyme that decomposes tropomyosin. Kim et al. (101) further investigated the allergenicity of *saeujeot* at various temperatures (25, 15, and 5°C) and salt concentrations (25, 15, and 10%). They showed that the binding ability of tropomyosin decreased faster at higher temperature and lower salt concentration. During fermentation, the allergenicity of *saeujeot* increased initially as tropomyosin dissolves in salt, while it is subsequently decomposed by enzymes.

## Future Perspectives

The clinical features of seafood allergy in Asia are distinct from the rest of the world. It is important to acknowledge such differences and to adapt our management strategies accordingly. As illustrated in peanut allergy, the key allergenic components were different in populations with high and low prevalences of peanut allergy (102). In shellfish, tropomyosin might be the key allergen in western cohorts while it is important to include other allergens such as troponin C and fatty acid-binding protein in Asians (62, 63). Fish collagen or other heat-labile proteins such as enolase and aldolase would be useful in fish allergy diagnosis in regions where raw fish consumption is common. We have also learnt that shrimp cephalothorax and fish roe can be important allergen sources, and these extracts should be standardized and made available for the clinical diagnosis of seafood allergy. In this review, we illustrated how the diagnosis of seafood allergy can be improved by studying clinically relevant or diet-related species in patients with mono-sensitization to specific seafood species. The advancement of molecular allergology prompted the identification of novel or species-specific allergens that allows us to derive more comprehensive panels for component-resolved diagnosis. Novel diagnostic platforms such as basophil activation test or EXiLE assays can also be adopted to test in-house allergen extracts in a standardized manner (94). Nonetheless, there are knowledge gaps over the prevalence and natural history of seafood allergy in Asian adults as well as the roles that dust mites and other environmental factors play in primary allergen sensitization. More challenge-based studies are also needed to identify novel seafood allergens in this region. These new scientific evidences will ultimately improve our clinical management strategies for seafood allergy.

## Author Contributions

CW and NL prepared the manuscript. AL, GW, and TL critically revised the manuscript. All authors contributed to manuscript revision, read, and approved the submitted version.

## Conflict of Interest

The authors declare that the research was conducted in the absence of any commercial or financial relationships that could be construed as a potential conflict of interest.
